# Osteosarcopenia, Osteoporosis, and Sarcopenia in Liver Cirrhosis: Prevalence, Predictors, and Prognostic Significance of IGF-1 Deficiency

**DOI:** 10.3390/jcm15072534

**Published:** 2026-03-26

**Authors:** Tanja Glamočanin, Tanja Veriš Smiljić, Marina Vukčević, Željka Savić, Renata Tamburić, Goran Bokan, Milan Kulić, Nenad Lalović, Nemanja Lazendić, Bojan Joksimović, Dario Djukić, Alma Prtina, Dajana Nogo-Živanović

**Affiliations:** 1 Internal Medicine Clinic, Department of Gastroenterology and Hepatology, University Clinical Center of the Republic of Srpska, 78000 Banja Luka, Bosnia and Herzegovina; tanja.glamocanin@kc-bl.com (T.G.); tanja.veris@kc-bl.com (T.V.S.); renata.tamburic@kc-bl.com (R.T.); goran.bokan@kc-bl.com (G.B.); 2 Faculty of Medicine, University of Banja Luka, 78000 Banja Luka, Bosnia and Herzegovina; nemanjalazendic@yahoo.com (N.L.); alma.prtina@med.unibl.org (A.P.); 3 Clinic of Radiology, University Clinical Centre of the Republic of Srpska, 78000 Banja Luka, Bosnia and Herzegovina; 4 Internal Medicine Clinic, Department of Rheumatology, University Clinical Center of the Republic of Srpska, 78000 Banja Luka, Bosnia and Herzegovina; marina.vukcevic@kc-bl.com; 5 Clinic for Gastroenterology and Hepatology, Clinical Centre of Novi Sad, 21000 Novi Sad, Serbia; zeljka.savic@mf.uns.ac.rs; 6 Faculty of Medicine Novi Sad, University of Novi Sad, 21000 Novi Sad, Serbia; 7 Faculty of Medicine Foča, University of East Sarajevo, 73300 Foča, Bosnia and Herzegovina; kulicmilan62@gmail.com (M.K.); nenad.lalovic@gmail.com (N.L.); bojannjoksimovic@gmail.com (B.J.); 8 Department for Rehabilitation of Patients with Chronic Non-Communicable Diseases, Institute for Physical Medicine and Rehabilitation and Orthopedic Surgery “Dr Miroslav Zotović”, 78000 Banja Luka, Bosnia and Herzegovina; 9 ASA Hospital, 71000 Sarajevo, Bosnia and Herzegovina

**Keywords:** liver cirrhosis, osteosarcopenia, sarcopenia, osteoporosis, IGF-1, mortality

## Abstract

**Background/Objectives:** Sarcopenia (SP) and osteoporosis (OP) are common yet underrecognized complications of liver cirrhosis, contributing to increased morbidity and mortality. Their coexistence, termed osteosarcopenia (OS), represents a compounded musculoskeletal impairment. Insulin-like growth factor 1 (IGF-1), synthesized in the liver, has been implicated in muscle and bone metabolism. This study aimed to assess the prevalence and association of laboratory and clinical parameters with SP, OP, and OS in cirrhotic patients, with a focus on IGF-1 deficiency and their impact on mortality. **Methods:** This cross-sectional study included 100 cirrhotic patients at a tertiary center. Sarcopenia was diagnosed using CT-derived L3 skeletal muscle index and osteoporosis via the DEXA scan. IGF-1 levels and metabolic parameters were measured. Multivariate logistic regression identified laboratory and clinical factors associated with musculoskeletal complications. However, due to the cross-sectional design, causal relationships could not be inferred. **Results:** SP, OP, and OS were present in 41%, 22%, and 11% of patients, respectively. IGF-1 levels were significantly lower in patients with SP, OP, and OS (*p* < 0.05) and were independently associated with increased risk of SP (OR = 1.797, *p* = 0.006), OP (OR = 1.873, *p* = 0.045), and OS (OR = 2.326, *p* = 0.003). Mortality rates were significantly higher among patients with OS (72.7%), OP (77.3%), and SP (56.1%). OS conferred the highest adjusted mortality risk (OR = 2.739, *p* = 0.009), followed by SP (OR = 2.278, *p* = 0.015) and OP (OR = 1.958, *p* = 0.036). **Conclusions:** Musculoskeletal complications are highly prevalent and predictive of mortality in cirrhosis. IGF-1 deficiency is a strong independent biomarker for SP, OP, and OS. Routine screening and early intervention targeting IGF-1 pathways and nutrition may improve outcomes in this population.

## 1. Introduction

Liver cirrhosis is a chronic irreversible liver disorder marked by fibrosis and structural distortion due to long-standing liver damage. In 2019, cirrhosis accounted for approximately 2.4% of deaths worldwide [1,2]. Widely applied prognostic tools like the Child–Turcotte–Pugh (CTP) and Model for End-Stage Liver Disease (MELD) scores are essential for estimating mortality risk in patients with cirrhosis [3]. In individuals with cirrhosis, the decline in functional liver capacity contributes to numerous complications, notably malnutrition and the onset of sarcopenia (SP) [4]. Protein–energy malnutrition, hyperammonemia, reduced levels of anabolic hormones such as insulin-like growth factor 1 (IGF-1), and increased levels of inflammatory cytokines contribute to an imbalance between protein synthesis and degradation, which results in SP [5].

SP, characterized by progressive loss of skeletal muscle mass, is a prevalent (22% to 55%) yet frequently underdiagnosed complication in patients with liver cirrhosis, adversely impacting survival rates [6,7] and the risk of cirrhosis-related complications, including ascites and hepatic encephalopathy (HE) [8]. Furthermore, SP roughly doubles the risk of mortality in individuals with cirrhosis [9]. Osteoporosis (OP), marked by a reduction in bone mass and the deterioration of bone microstructure, is also a frequent complication in patients with cirrhosis [10]. The frequent coexistence of SP and OP led to the term “osteosarcopenia” (OS) introduced in 2009 [11]. A recent retrospective study (2023) determined that OS is an independent predictor of poor survival in cirrhotic patients, with significantly lower 1-, 3-, and 5-year survival compared to those without the condition [12]. High overall prevalence of OS in cirrhotic patients (16–20%) is clinically critical because it predicts mortality and hepatic decompensation, and it increases healthcare burden in this population [10,12,13].

Low levels of IGF-1 are commonly observed in patients with liver cirrhosis, and a further decline in IGF-1 concentration has been associated with disease progression [14]. This reduction is also linked to malnutrition, which contributes to the development of both OP and SP, and it is an independent predictor of frailty, poorer functional status, and increased waitlist mortality in patients awaiting liver transplantation [15,16]. Recent findings have demonstrated a strong association between low IGF-1 levels and increased prevalence of SP and OP, as well as a higher incidence of complications such as ascites and HE [17]. Previous research has examined the relationship between circulating IGF-1 levels and SP using anthropometric assessments, which are limited in decompensated cirrhosis [18,19]. However, no studies have evaluated the correlation between various risk factors to date, especially IGF-1 levels and the coexistence of OP, SP, and OS in cirrhotic patients using the L3 skeletal muscle index (L3-SMI)—a more objective and reliable method. Therefore, this study aimed to assess the prevalence and predictors of SP, OP, and OS in cirrhotic patients with a focus on IGF-1 deficiency, and the impact of these conditions on mortality.

## 2. Materials and Methods

### 2.1. Study Design

The research was conducted as a cross-sectional study on the territory of the Bosnia and Herzegovina in the University Clinical Center Republic of Srpska (UCCSRS). The study was carried out after taking informed written consent from all individuals and approval of the Ethics Committee of the UCSRS (approval number: 01-19-79-2/20), from 1 June 2020 to 1 June 2024.

### 2.2. Patients

The study included patients with liver cirrhosis of any etiology, older than 18 years, whose diagnosis was confirmed based on laboratory parameters and abdominal ultrasonography (US). In order to confirm the diagnosis, abdominal computed tomography (CT) scan was performed. Patients with malignancies, prolonged corticosteroid use, and cardiac or pulmonary dysfunctions were excluded from the study.

### 2.3. Laboratory and Clinical Parameters

Clinical parameters included data obtained from the electronic medical records, including patient age, sex, etiology of cirrhosis, and the presence of ascites and HE. Additional data were extracted during physical examination of patients, and body mass index (BMI) (kg/m^2^) was measured. Routine laboratory analyses such as white blood cell (WBC), red blood cell (RBC), hemoglobin (Hgb), and platelet (PLT) count, serum proteins and albumins, total bilirubin (TB), conjugated bilirubin (CB), aspartate aminotransferase (AST), alanine aminotransferase (ALT), gamma-glutamyl transferase (GGT), alkaline phosphatase (ALP), international normalized ratio (INR), vitamin D, parathyroid hormone (PTH), ammonia, total calcium (Ca), ionized Ca (iCa), phosphate (P), urea, and creatinine were performed for all subjects, using the enzyme chemiluminescence method on the “DXI-600, Beckam Coulter” analyzer (Beckman Coulter, Inc., 250 S. Kraemer Blvd., Brea, CA, USA). The serum IGF-1 levels were determined using an enzyme immunoassay (EIA), with all procedures carried out in accordance with the manufacturer’s instructions in the biochemical laboratory in UCSRS. The degree of ascites was defined by the International Club of Ascites (ICA) guidelines [20]. HE was defined by the West Haven grading system [21]. The CTP (class A, B and C) and the MELD score (ranging from 6 to 40) were used to assess the liver functional status [3,22,23].

### 2.4. Diagnosis of Sarcopenia and Osteoporosis

After establishing the diagnosis, abdominal multislice CT scan was performed. The L3-SMI was automatically calculated using computer software (BMI_CT, Matlab version R2010) [24]. The diagnostic values were taken from oncology population patients, where SP is defined as <38.5 cm/m^2^ for women and <52.4 cm/m^2^ for men [25]. Although the cut-offs were originally established in oncology populations, they have been widely used and validated in patients with liver cirrhosis in previous studies and are considered reliable for assessing sarcopenia in this context [26,27]. Skeletal muscle tissues were identified on CT images using Hounsfield unit (HU) values from −29 to +150 [28]. Osteoporosis was diagnosed using osteodensitometry (DEXA) according the World Health Organization (WHO) criteria (T-score ≤ −2.5) [29]. For the purpose of this study, the patients without osteoporosis (normal report and osteopenia) were combined into one group. OS was defined as the coexistence of SP and OP [11].

### 2.5. Statistical Analysis

To detect a statistically significant difference among subject groups at a *p* < 0.05 significance level with 80% study power, the study included 100 patients. The sample size was calculated using the G*Power program version 3.1, with a chi square (χ^2^) test. Group frequencies were compared using χ^2^ test, and independent *t* test or Mann–Whitney test was used to test the differences in mean values. An association between various risks factors for the occurrence of OS, OP, and SP were assessed using the adjusted odds ratio (OR) with 95% confidence intervals (95% CI) using binary multivariate logistic regression analysis. The regression models were adjusted using univariately significant variables. The data were analyzed using the statistical software SPSS, version 24 (Chicago, IL, USA). The results were expressed as mean value ± standard deviation (SD), and *p*-value less than 0.05 was considered statistically significant.

## 3. Results

**Figure 1:**
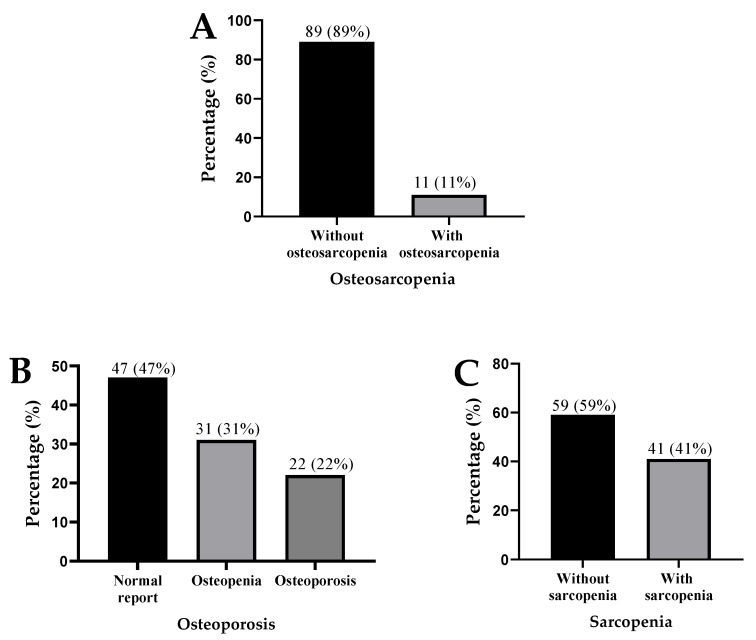
Presence of osteosarcopenia (**A**), osteoporosis (**B**), and sarcopenia (**C**) in patients with liver cirrhosis.

Out of 100 patients, 11% had OS, 22% were with OP, and 41% had SP (Figure 1A–C).

The mean age of patients was 55.05 ± 10.64 years, and 88% of the participants were male. The patients with OS had a significantly lower BMI compared to those without OS (*p* < 0.001). At the time of the examination, only 5% of patients had preserved liver function (CTP score A), 49% had moderate liver dysfunction (score B), and 46% had severe liver dysfunction (score C). The average MELD score was 17.04 ± 6.58. Alcohol-related liver disease was the underlying cause of cirrhosis in 88% of patients. The remaining 12% had cirrhosis due to hepatitis B, nonalcoholic steatohepatitis (NASH), or primary biliary cholangitis (PBC). Grade 3 ascites was observed in 48% of patients, and grade 3 HE was present in 6% of patients. The patients with OS had higher levels of ALP (*p* = 0.009) and PTH (*p* = 0.023) and lower levels of total Ca (*p* = 0.044) and iCa (*p* = 0.006) compared to those without OS. The mortality rate during the study period was higher among the patients with OS (72.7% vs. 39.3%) (*p* = 0.035) (Table 1).

The patients with SP were predominantly male (*p* = 0.014) and younger (*p* = 0.048), and they had lower BMI values (*p* = 0.008) and a shorter duration of cirrhosis (*p* = 0.043) than non-SP patients. Patients with SP had higher WBC counts (*p* = 0.020) and AST levels (*p* = 0.005), along with lower RBC counts (*p* = 0.013) and iCa (*p* = 0.009) and IGF-1 levels (*p* = 0.019). The mortality rate was also higher among the SP patients compared to the non-SP patients (56.1% vs. 33.9%; *p* = 0.027). The patients with OP had lower levels of BMI (*p* = 0.031), vitamin D (*p* = 0.003), total Ca (*p* = 0.029), iCa (*p* = 0.014), P (*p* = 0.046), and IGF-1 (*p* < 0.001). In contrast, they had higher levels of ALP (*p* = 0.003) and PTH (*p* = 0.006). Mortality was also higher in the OP group (77.3% vs. 33.3%; *p* < 0.001) (Table 2).

The multivariate analysis showed that factors associated with OS (OR = 0.430; *p* = 0.005), OP (OR = 0.534; *p* = 0.009), and SP (OR = 0.691; *p* = 0.001) were lower values of IGF-1. Furthermore, OS (OR = 1.720; *p* = 0.021), OP (OR = 1.438; *p* = 0.026), and SP (OR = 1.221; *p* < 0.001) were significantly associated with mortality, which means that the presence of these conditions was independently associated with lower values of IGF-1 and mortality in patients with liver cirrhosis in models where univariately significant variables were included (Table 3).

## 4. Discussion

This study demonstrates that musculoskeletal complications are highly prevalent in patients with liver cirrhosis, with SP (41%), OP (22%), and OS (11%) affecting a substantial proportion of our cohort. These prevalence rates are consistent with previously reported ranges in cirrhotic populations: SP in 22–55%, OP in 20–30%, and OP in 16–19% [5,6,10,12,13]. Our OS prevalence of 11% is somewhat lower than the 16.3% reported by Saeki et al. in a 2023 retrospective study of 135 cirrhotic patients and the 19% observed in their 2019 cohort [10,12]. This discrepancy may reflect differences in diagnostic criteria, etiology distribution, or disease severity between populations. Our use of CT-based L3-SMI and DEXA, objective and gold-standard diagnostic tools, likely enhanced the accuracy of detection in this vulnerable group [24]. The use of L3-SMI by CT scan in our methodology offered a reliable assessment of muscle mass, especially relevant in cirrhosis where ascites and edema limit the utility of anthropometric measures. Studies by Zeng et al. and Kim et al. confirm L3-SMI as a reliable predictor of survival and complications in cirrhosis, underscoring its clinical value [8,24].

The principal finding of this study is the association between low IGF-1 levels and all three musculoskeletal conditions confirmed by univariate and multivariate analysis. IGF-1, predominantly synthesized in the liver, plays a key role in the skeletal muscle and bone metabolism [14]. Its decline in cirrhosis has been associated with SP and OP, correlating with muscle atrophy, reduced bone mineral density, and increased risk of cirrhosis-related complications [17,19]. In our multivariate regression models adjusted for laboratory and clinically relevant covariates, IGF-1 remained positively associated with OS, OP, and SP. These findings align closely with the recent work by Kaur et al. (2025), who reported significantly lower IGF-1 levels in cirrhotic patients with SP (*p* < 0.001) and identified IGF-1 as an independent predictor of both muscle loss and mortality in a cohort of 220 decompensated cirrhotic patients [17]. In their multivariate analysis, each 10 ng/mL decrease in IGF-1 was associated with a 1.4-fold increased risk of SP, comparable to the effect size observed in our study. Similarly, Saeki et al. (2020) demonstrated that low IGF-1 combined with low branched-chain amino acid levels was associated with SP and slow gait speed in 128 cirrhotic patients, with odds ratios (OR = 2.81 for SP in patients with both deficiencies) that parallel our findings [18]. The consistency of these results across different populations and geographic regions strengthens the evidence, linking IGF-1 deficiency to musculoskeletal deterioration in cirrhosis. However, due to the cross-sectional nature of our study, the association between low IGF-1 levels and SP, OP, and OS cannot establish whether reduced IGF-1 plays a causal role in the development of these conditions or simply represents a biomarker of advanced liver dysfunction.

Beyond IGF-1, our univariate analysis identified several metabolic disturbances associated with these conditions. Patients with OS and OP exhibited lower BMI, elevated ALP and PTH, and patients with OP reduced Ca, vitamin D and phosphate—while those with SP showed a more restricted pattern characterized primarily by lower BMI and reduced iCa, indicating that this hormonal deficiency likely does not operate independently and suggests that systemic metabolic disturbances—driven by malnutrition and secondary hyperparathyroidism—play synergistic roles in musculoskeletal deterioration [17]. Significantly lower BMI in our patients with OS and OP underscores the importance of body composition—not just body weight—as a determinant of health outcomes in cirrhosis [9]. Saeki et al. (2019) reported similar patterns, with significantly lower BMI and higher ALP and PTH, with reduced calcium in 93 cirrhotic patients with OS compared to those without, although their cohort showed more pronounced vitamin D deficiency (mean 5.2 ng/mL in OS patients) than ours [10]. Bunchorntavakul and Reddy (2020) documented that vitamin D deficiency affects 64–93% of cirrhotic patients and correlates with reduced bone mass and poor neuromuscular coordination [7]. This vitamin D deficiency is consistent with the low mean vitamin D levels we observed across all groups (range 6.95–10.60 ng/mL), though only OP patients showed significantly lower levels compared to their non-osteoporotic counterparts. This discrepancy suggests that while vitamin D depletion is nearly universal in advanced cirrhosis, its association with bone disease may be more pronounced in populations with greater nutritional compromise or differing etiology profiles [30,31]. The consistency of these abnormalities across studies underscores the importance of secondary hyperparathyroidism and vitamin D deficiency in the pathogenesis of bone disease in cirrhosis [2]. In biochemical analyses, patients with OS and OP showed significantly elevated ALP and PTH levels, indicative of high bone turnover. Concurrently, reductions in total and iCa and phosphate were observed. These abnormalities reflect secondary hyperparathyroidism and disrupted bone remodeling, both well-described complications of chronic liver disease [2]. However, in our multivariate analysis, only IGF-1 retained statistical significance across all three conditions. This contrasts partially with findings from Kaur et al., where PTH remained independently associated with OP in their final models (OR = 1.03 per unit increase, *p* = 0.04) [17], possibly reflecting differences in sample size or disease severity between studies. The loss of statistical significance for other metabolic parameters after adjustment in our models suggests that IGF-1 may integrate multiple metabolic disturbances or represent a more proximal determinant of musculoskeletal health in this population. An unexpected observation was the relatively younger age of sarcopenic patients to those without SP in our cohort. This contrasts with the typical age-related trajectory of muscle loss observed in general populations but is consistent with findings in predominantly alcoholic cirrhosis cohorts. Similarly, Zeng et al. (2021), in a multicenter Chinese study of 386 cirrhotic patients, found that SP was not age-dependent and occurred earlier in patients with alcoholic etiology, with mean age of sarcopenic patients (54.2 ± 9.8 years) nearly identical to our cohort [8]. These findings can be explained by increased muscle wasting, which often begins earlier due to poor dietary intake, alcohol-induced myopathy, and systemic catabolism [8]. The high proportion of alcoholic liver disease (88%) in our cohort likely contributes to this pattern and should be considered when interpreting these results.

Mortality was significantly elevated across all three conditions, with the highest rate in OP patients (77.3%), followed by OS (72.7%) and SP (56.1%). After multivariate adjustment for IGF-1, BMI, and relevant metabolic parameters, OS conferred the highest association with mortality (OR = 1.720; *p* = 0.021), followed by OP (OR = 1.438; *p* = 0.026) and SP (OR = 1.221; *p* < 0.001). These findings reinforce the meta-analysis by Tantai et al. (2022), which included 43 studies with 12,366 cirrhotic patients and demonstrated that SP nearly doubles mortality risk (pooled OR = 1.92; *p* < 0.05) [9]. Our observed OR of 1.221 for SP is lower but close to this estimate, and our findings expand this risk stratification when OP and OS were included. Notably, Saeki et al. (2023) reported that OS was an independent predictor of poor survival in 135 cirrhotic patients, with 1-, 3-, and 5-year survival rates of 79.2%, 54.1%, and 33.4% in OS patients compared to 96.9%, 81.5%, and 70.9% in those without the condition (*p* < 0.001) [12]. While our cross-sectional design precludes survival analysis, the strong association between OS and mortality aligns with their findings and supports the clinical significance of this combined phenotype [12]. Although in our study OP showed the highest rate of mortality, after adjusting for IGF-1, BMI, and calcium phosphate levels, its direct effect on mortality appears weaker than that of OS. Unlike SP, OP and OS more directly impair function, immunity, and metabolic reserve, leading to worse short-term outcomes [5,7,8,17]. Moreover, this gradient of increasing mortality from OP alone to SP alone to combined OS suggests that concurrent muscle and bone loss exerts a synergistic effect on survival [10,18,23]. This pattern is consistent with findings from Clynes et al. (2021), who reviewed evidence that individuals with both conditions have worse outcomes than those with either condition alone, with OR for mortality ranging from 1.5 to 3.0 across different populations [32]. Similarly, in the specific context of cirrhosis, Saeki et al. (2023) found that OS was more strongly associated with mortality than either condition individually [12].

From a pathophysiological standpoint, chronic inflammation, metabolic disturbances, and IGF-1 deficiency disrupt muscle and bone homeostasis, explaining why combined SP and OP in OS leads to worse outcomes than either condition alone [25,28,32]. An additional mechanism increasingly recognized in cirrhosis-related SP involves the spleen–liver axis. Spleen enlargement (hypersplenism) is common in cirrhosis and directly linked to muscle wasting through altered inflammatory signaling, nutrient sequestration, and elevated myostatin expression. Studies have demonstrated that interventions targeting the enlarged spleen—such as splenectomy or splenic artery embolization (SAE)—can prevent or improve SP by reducing myostatin levels and enhancing muscle regeneration. These spleen-focused approaches offer new hope for managing this debilitating condition, as the spleen–liver axis contributes to the progression of secondary SP through multiple interconnected mechanisms involving portal hypertension, systemic inflammation, and metabolic dysregulation [33]. Given the association of IGF-1 with OS, OP, and SP, this hormone holds promise not only as a biomarker but potentially as a therapeutic target. IGF-1 supplementation, though still experimental in cirrhosis, has shown favorable effects on muscle regeneration and bone formation in other populations [34].

Several limitations warrant consideration. First, the cross-sectional design precludes causal inference; the associations we report, while statistically robust, cannot establish directionality. Second, the relatively small sample size—particularly the OS group (*n* = 11)—limits statistical power for some subgroup analyses and affects the precision of multivariate estimates, as reflected in the wide confidence intervals for some mortality associations. Future studies with larger cohorts are needed to validate our findings. Third, the predominance of alcoholic liver disease (88%) limits generalizability to other etiologies such as viral hepatitis or NASH; studies in more diverse populations are needed to validate our findings. Additionally, the wide CI observed for certain variables in our multivariate models—particularly in the OS subgroup—reflects the limited sample size and variability inherent to this population. These findings should therefore be interpreted with caution and confirmed in larger prospective studies.

Our study includes a relatively small sample size, particularly in the OS group, which may reduce the statistical power of the logistic regression model. The predominance of alcoholic liver disease (88%) in our cohort limits the generalizability of our findings to other etiologies, such as viral hepatitis or NASH. Therefore, our results should be validated in more diverse populations. Fourth, while CT-based L3 skeletal muscle index is considered objective, the cut-off values were derived from oncology populations (women < 38.5 cm^2^/m^2^ and men < 52.4 cm^2^/m^2^), although these thresholds have been validated in cirrhosis [25,26,27]. Finally, we did not assess physical performance measures (e.g., gait speed or handgrip strength), which are incorporated in contemporary SP definitions and might provide additional functional insights beyond muscle mass alone [31]. Despite these limitations, the study has notable strengths. The use of objective, gold-standard diagnostic methods (CT for muscle mass and DEXA for bone density) ensures accurate classification and overcomes the limitations of anthropometric assessment in cirrhotic patients. The comprehensive laboratory evaluation allowed the exploration of multiple metabolic pathways simultaneously. The inclusion of OS as a distinct category enabled the evaluation of the combined effect of muscle and bone loss, which remains understudied in cirrhosis. Our findings contribute to the growing evidence base supporting routine screening for musculoskeletal complications in this vulnerable population.

## 5. Conclusions

Our findings emphasize that OS, OP, and SP are prevalent, interrelated, and independently predictive of mortality in patients with liver cirrhosis. Low IGF-1 emerges as a unifying biomarker, linking hepatic dysfunction to systemic musculoskeletal deterioration. The use of CT-derived muscle indices and DEXA-based bone assessments allowed for precise classification, revealing that nearly half of all patients are affected by at least one of these debilitating conditions. Clinicians should proactively screen for SP and OP in cirrhotic patients, especially those with low BMI, high ALP/PTH, or low IGF-1. Interventions aimed at improving muscle and bone health—whether hormonal, nutritional, or physical—are urgently needed. Future research should prioritize longitudinal studies to assess the impact of targeted IGF-1-based therapies on reversing or mitigating these conditions with the goal of improving survival and quality of life in patients facing the systemic consequences of cirrhosis.

## Figures and Tables

**Table 1:** Comparison of socio-demographic, clinical, and laboratory characteristics in cirrhosis patients with and without osteosarcopenia.

Variables	Without OS (*n* = 89, 89%)		OS (*n* = 11, 11%)		Total (*n* = 100, 100%)		*p* *
	*n*	%	*n*	%	*n*	%	
Male gender	78	87.6	10	90.9	88	88.0	0.753 *
Age (M ± SD)	55.05 ± 10.99		55.00 ± 7.60		55.05 ± 10.64		0.987 **
30 to 50 years	28	31.5	3	27.3	31	31.0	0.777 *
51 to 79 years	61	68.5	8	72.7	69	69.0	
BMI (kg/m^2^) (M ± SD)	28.20 ± 4.65		22.05 ± 5.79		25.25 ± 4.92		**<0.001** **
CTP score							
A	5	5.6	0	0.0	5	5.0	0.655 *
B	44	49.4	5	45.5	49	49.0	
C	40	44.9	6	54.5	46	46.0	
MELD score	16.84 ± 6.57		18.63 ± 6.75		17.04 ± 6.58		0.397 **
Etiology							
Alcoholism	78	87.6	10	90.9	88	88.0	
Hepatitis B	2	2.2	0	0.0	2	2.0	
Alcoholism + hepatitis B	3	3.4	0	0.0	3	3.0	0.360 *
NASH	5	5.6	0	0.0	5	5.0	
PBC	1	1.1	1	9.1	2	2.0	
Cirrhosis duration (M ± SD)	1.73 ± 2.48		0.89 ± 1.62		1.64 ± 2.41		0.231 *
Newly diagnosed	48	53.9	8	72.7	56	56.0	0.236 *
More than 1 year	41	46.1	3	27.3	44	44.0	
Ascites							
Grade 0	12	13.5	1	9.1	13	13.0	
Grade 1	7	7.9	9	0.0	7	7.0	0.631 *
Grade 2	27	30.3	5	45.5	32	32.0	
Grade 3	43	48.3	5	45.5	48	48.0	
HE							
Grade 0	49	55.1	4	36.4	53	53.0	
Grade 1	15	16.9	3	27.3	18	18.0	0.675 *
Grade 2	20	22.5	3	27.3	23	23.0	
Grade 3	5	5.6	1	9.1	6	6.0	
Laboratory analyses (M ± SD)							
Hematology							
WBC (3.71–10.67 × 10^9^/L)	7.26 ± 3.71		8.27 ± 2.91		7.37 ± 3.64		0.410 **
RBC (3.87–5.68 × 10^12^/L)	3.47 ± 0.66		3.34 ± 0.48		3.46 ± 0.64		0.540 **
Hemoglobin (120–175 g/L)	107.22 ± 28.69		109.19 ± 19.44		107.42 ± 27.80		0.841 ^#^
PLT (150–450 × 10^9^/L)	113.31 ± 68.33		134.40 ± 14.27		115.51 ± 72.46		0.387 ^#^
Liver function tests							
ALT (8–48 IU/L)	51.65 ± 51.98		39.72 ± 23.81		50.34 ± 49.73		0.456 ^#^
AST (7–55 IU/L)	105.71 ± 86.50		112.72 ± 52.08		106.49 ± 83.24		0.794 ^#^
GGT (6–61 U/L)	341.27 ± 222.15		370.18 ± 181.36		344.48 ± 199.40		0.867 ^#^
ALP (54–214 IU/L)	120.61 ± 73.63		190.36 ± 63.21		516.9 ± 67.32		**0.009** ^#^
TB (3–30 μmol/L)	97.60 ± 115.38		90.60 ± 72.06		96.83 ± 102.11		0.679 ^#^
CB (0–5 μmol/L)	58.58 ± 80.05		51.81 ± 50.49		57.83 ± 77.19		0.899 ^#^
Total proteins (66–81 g/L)	58.44 ± 22.47		64.20 ± 5.89		59.16 ± 21.16		0.576 **
Albumins (30–50 g/L)	29.52 ± 6.62		27.63 ± 4.31		29.32 ± 6.42		0.360 **
INR (0.80–1.20)	1.52 ± 0.52		1.50 ± 0.37		1.52 ± 0.50		0.924 **
Kidney function tests							
Urea (2.8–7.2 μmol/L)	5.87 ± 3.97		6.21 ± 2.98		5.91 ± 3.86		0.384 **
Creatinine (58–110 μmol/L)	80.19 ± 35.44		82.38 ± 39.46		80.43 ± 35.70		0.437 ^#^
Bone metabolism markers							
Vitamin D (30–50 ng/mL)	9.98 ± 5.97		8.38 ± 5.58		9.80 ± 5.92		0.401 **
PTH (15–65 pg/mL)	30.64 ± 15.48		65.18 ± 90.08		34.44 ± 33.92		**0.023** ^#^
Total Ca (2.1–2.6 mmol/L)	2.20 ± 0.16		2.02 ± 0.09		2.09 ± 0.15		**0.044** **
iCa (1.1–1.3 mmol/L)	1.26 ± 0.07		1.12 ± 0.03		1.18 ± 0.07		**0.006** **
P (0.79–1.42 mmol/L)	1.02 ± 0.22		0.96 ± 0.16		1.01 ± 0.22		0.450 **
Other metabolic parameters							
Ammonia (10–47 μmol/L)	56.08 ± 38.68		58.65 ± 16.21		56.36 ± 36.82		0.829 ^#^
IGF-1 (85–245 ng/mL)	56.31 ± 36.03		29.25 ± 17.44		53.33 ± 22.39		**0.006** ^#^
Mortality (yes)	35	39.3	8	72.7	43	43.0	**0.035** *

OS—osteosarcopenia, CTP—Child–Turcotte–Pugh, MELD—Model for End-Stage Liver Disease, NASH—nonalcoholic steatohepatitis, PBC—primary biliary cholangitis, HE—hepatic encephalopathy, WBCs—white blood cell, RBCs—red blood cells, PLTs—platelets, ALT—alanin aminotransferase, AST—aspartat aminotransferase, GGT—gamma glutamyi transferase, ALP—alkaline phosphatase, TB—total bilirubin, CB—conjugated bilirubin, PTH—parathyroid hormone, Ca—calcium, iCa—ionizied calcium, P—phosphate, INR—international normalized ratio, IGF-1—insulin-like growth factor 1, M—mean, SD—standard deviation; *p*—statistical significance was measured by * χ^2^—chi square test, ** independent *t* test, or ^#^ Mann–Whitney U test; significant values are bolded.

**Table 2:** Comparison of socio-demographic, clinical, and laboratory characteristics in cirrhosis patients with and without osteoporosis and sarcopenia.

Variables	Without OP (*n* = 78, 78%)		OP (*n* = 22, 22%)		*p* *	Without SP (*n* = 71, 67%)		SP (*n* = 35, 33%)		*p* *
	*n*	%	*n*	%		*n*	%	*n*	%	
Male gender	71	91.0	17	77.3	0.080 *	48	81.4	40	97.6	**0.014** *
Age (M ± SD)	54.43 ± 11.18		57.22 ± 8.30		0.279 **	56.79 ± 10.02		52.53 ± 11.12		**0.048** **
30 to 50 years	27	34.6	4	18.2	0.141 *	15	25.4	16	39.0	0.148 *
51 to 79 years	51	65.4	18	81.8		44	74.6	25	61.0	
BMI (kg/m^2^) (M ± SD)	28.51 ± 4.65		22.40 ± 4.26		**0.031** **	29.66 ± 3.33		20.85 ± 2.19		**0.008** **
CTP score										
A	5	6.4	0	0.0	0.242 *	4	6.8	1	2.4	0.196 *
B	40	51.3	9	40.9		32	54.2	17	41.5	
C	33	42.3	13	59.1		23	39.0	23	56.1	
MELD score	16.60 ± 6.74		18.59 ± 5.86		0.182 **	16.08 ± 6.09		18.41 ± 7.07		0.113 **
Etiology										
Alcoholism	68	87.2	20	90.9		52	88.1	36	87.8	
Hepatitis B	2	2.6	0	0.0		0	0.0	2	4.9	
Alcoholism + hepatitis B	3	0.0	0	0.0	0.061 *	2	3.4	1	2.4	0.416 *
NASH	5	6.4	0	0.0		4	6.8	1	2.4	
PBC	0	0.0	2	9.1		1	1.7	1	2.4	
Cirrhosis duration (M ± SD)	1.66 ± 2.43		1.56 ± 2.38		0.881 **	2.03 ± 2.61		1.08 ± 1.99		**0.043** **
Newly diagnosed	42	53.8	14	63.6	0.414 *	30	50.8	26	63.4	0.213 *
More than 1 year	36	46.2	8	36.4		29	49.2	15	36.6	
Ascites										
Grade 0	12	15.4	1	4.5		11	18.6	2	4.9	
Grade 1	6	7.7	1	4.5	0.529 *	4	6.8	3	7.3	0.077 *
Grade 2	24	30.8	8	36.4		14	23.7	18	43.9	
Grade 3	36	46.2	12	54.5		30	50.8	18	43.9	
HE										
Grade 0	44	56.4	9	40.9		35	59.3	18	43.9	
Grade 1	13	16.7	5	22.7	0.615 *	9	15.3	9	22.0	0.506 *
Grade 2	17	21.8	6	27.3		12	20.3	11	26.8	
Grade 3	4	5.1	2	9.1		3	5.1	3	7.3	
Laboratory analyses (M ± SD)										
Hematology										
WBC (3.71–10.67 × 10^9^/L)	7.43 ± 3.83		7.16 ± 2.94		0.989 **	6.68 ± 3.27		8.45 ± 3.96		**0.020** **
RBC (3.87–5.68 × 10^12^/L)	3.48 ± 0.65		3.38 ± 0.63		0.978 **	3.59 ± 0.67		3.26 ± 0.55		**0.013** **
Hgb (120–175 g/L)	108.03 ± 25.90		105.24 ± 34.37		0.794 ^#^	106.80 ± 32.37		108.36 ± 19.18		0.789 ^#^
PLT (150–450 × 10^9^/L)	115.96 ± 65.58		113.90 ± 94.97		0.390 ^#^	112.27 ± 71.22		120.44 ± 74.99		0.592 ^#^
Liver function tests										
ALT (8–48 IU/L)	48.0 ± 45.88		56.50 ± 62.35		0.184 ^#^	52.69 ± 58.66		46.95 ± 33.33		0.573 ^#^
AST (7–55 IU/L)	102.33 ± 87.37		121.22 ± 66.25		0.063 ^#^	101.08 ± 99.38		114.26 ± 52.21		**0.005** ^#^
GGT (6–61 U/L)	339.94 ± 550.02		360.36 ± 502.54		0.085 ^#^	345.00 ± 90.12		343.75 ± 94.23		0.991 ^#^
ALP (54–214 IU/L)	115.05 ± 61.40		174.95 ± 128.91		**0.003** ^#^	121.43 ± 85.96		138.17 ± 82.01		0.333 ^#^
TB (3–30 cμmol/L)	97.37 ± 121.32		94.90 ± 65.69		0.152 ^#^	79.13 ± 85.98		122.30 ± 136.9		0.056 ^#^
CB (0–5 μmol/L)	58.36 ± 84.43		55.98 ± 44.13		0.194 ^#^	46.41 ± 58.29		74.27 ± 96.70		0.079 ^#^
Total proteins (66–81 g/L)	57.45 ± 23.76		64.30 ± 8.99		0.842 **	55.06 ± 25.29		65.31 ± 8.13		0.135 **
Albumins (30–50 g/L)	29.53 ± 6.15		28.54 ± 7.41		0.262 **	30.28 ± 6.88		27.92 ± 5.48		0.070 **
INR (0.80–1.20)	1.51 ± 0.54		1.55 ± 0.34		0.140 **	1.44 ± 0.38		1.63 ± 0.63		0.071 **
Kidney function tests										
Urea (2.8–7.2 μmol/L)	5.74 ± 3.68		6.51 ± 4.49		0.411 **	6.18 ± 3.79		5.53 ± 3.98		0.410 **
Creatinine (58–110 μmol/L)	78.57 ± 33.23		87.00 ± 43.59		0.330 ^#^	83.67 ± 36.64		75.76 ± 34.19		0.278^#^
Bone metabolism markers										
Vitamin D (30–50 ng/mL)	10.60 ± 6.06		6.95 ± 4.43		**0.010** **	10.48 ± 6.40		8.82 ± 5.07		0.170 **
PTH (15–65 pg/mL)	29.50 ± 15.88		51.95 ± 63.93		**0.006** ^#^	29.98 ± 15.76		40.85 ± 49.15		0.115 ^#^
Total Ca (2.1–2.6 mmol/L)	2.11 ± 0.16		1.42 ± 0.10		**0.029** **	2.10 ± 0.16		2.08 ± 0.15		0.553 **
iCa (1.1–1.3 mmol/L)	1.37 ± 0.07		1.05 ± 0.05		**0.014** **	1.28 ± 0.07		1.10 ± 0.07		**0.009** **
P (0.79–1.42 mmol/L)	1.03 ± 0.22		0.93 ± 0.19		**0.046** **	0.99 ± 0.21		1.03 ± 0.22		0.378 **
Other metabolic markers										
Ammonia (10–47 μmol/L)	56.34 ± 40.47		56.44 ± 19.98		0.151 ^#^	57.40 ± 43.08		54.83 ± 25.37		0.735 ^#^
IGF-1 (85–245 ng/mL)	60.70 ± 79.71		27.22 ± 22.24		**<0.001** ^#^	65.52 ± 20.15		35.79 ± 25.56		**0.019** ^#^
Mortality (yes)	26	33.3	17	77.3	**<0.001** ^#^	20	33.9	23	56.1	**0.027** *

OP—osteoporosis, SP—sarcopenia, CTP—Child–Turcotte–Pugh, MELD—Model For End-Stage Liver Disease, NASH—Nonalcoholic steatohepatitis, PBC—primary biliary cholangitis, HE—hepatic encephalopathy, WBCs—white blood cells, RBCs—red blood cells, Hgb—hemoglobin, PLTs—platelets, ALT—alanin aminotransferase, AST—aspartat aminotransferase, GGT—gamma glutamyi transferase, ALP—alkaline phosphatase, TB—total bilirubin, CB—conjugated bilirubin, PTH—parathyroid hormone, Ca—calcium, iCa—ionizied calcium, P—phosphate, INR—international normalized ratio, IGF-1—insulin-like growth factor 1, M—mean, SD—standard deviation; *p*—statistical significance was measured by * χ^2^—chi square test, ** independent *t* test, or ^#^ Mann–Whitney U test; significant values are bolded.

**Table 3:** Regression analysis of univariately significant variables as predictors of osteosarcopenia, osteoporosis, and sarcopenia in patients with liver cirrhosis hospitalized at the University Clinical Center Republic of Srpska.

Regression Models	Multivariate			
	B	Adjusted OR	95% CI	*p*
Osteosarcopenia				
Constant	10.002	2.315	/	0.372
BMI	−0.233	0.591	0.332–1.351	0.108
ALP	0.007	0.269	0.043–0.521	0.175
PTH	1.322	1.074	0.291–4.261	0.421
Total Ca	−0.425	0.831	0.388–1.257	0.722
iCa	−0.035	0.413	0.102–0.891	0.920
IGF-1	−0.844	0.430	0.246–0.751	**0.005**
Mortality	1.008	1.720	0.832–7.050	**0.021**
Osteoporosis				
Constant	9.238	1.220	/	0.490
BMI	−0.550	0.671	0.016–2.239	0.933
ALP	0.916	2.500	0.566–7.239	0.396
Vitamin D	−0.031	0.970	0.484–1.945	0.843
PTH	0.203	1.003	0.761–1.565	0.207
Total Ca	−0.772	0.462	0.130–1.649	0.491
iCa	−0.602	0.548	0.037–8.001	0.379
P	−0.957	0.399	0.095–2.147	0.483
IGF-1	−0.627	0.534	0.289–0.998	**0.009**
Mortality	0.672	1.438	1.009–3.518	**0.026**
Sarcopenia				
Constant	7.221	1.352	/	0.664
Male gender	0.916	2.508	0.763–6.487	0.926
Age	1.249	0.128	0.029–0.538	0.421
BMI	−0.077	0.372	0.103–0.552	0.639
Cirrhosis duration	−2.364	0.195	0.049–0.493	0.871
WBC	0.405	1.500	0.690–4.792	0.488
RBC	−0.364	0.511	0.281–1.256	0.209
AST	0.182	1.200	0.960–1.449	0.482
iCa	−2.430	0.305	0.091–1.028	0.727
IGF-1	−0.587	0.691	0.363–0.950	**0.001**
Mortality	0.823	1.221	0.692–2.834	**<0.001**

BMI—body mass index, WBCs—white blood cells, RBCs—red blood cells, AST—aspartat aminotransferase, ALP—alkaline phosphatase, PTH—parathyroid hormone, Ca—calcium, iCa—ionizied calcium, P—phosphate, IGF-1—insulin-like growth factor 1, B—unstandardized regression coefficient; adjusted OR—odds ratio; 95% CI—confidence interval, *p*—statistical significance; significant values are bolded.

## Data Availability

The data presented in this study are available on request from the corresponding author due to privacy and ethical restrictions.
